# Redefining Aging Without Family Support: Welfare Needs & Service Use Among Never-Married Older Women

**DOI:** 10.1093/geroni/igaf122.2494

**Published:** 2025-12-31

**Authors:** Shiau-Fang Chao, Yueh-Tzu Wang, Chien-Chou Hou, Ju-Ping Lin

**Affiliations:** National Taiwan University, Taipei, Taipei, Taiwan (Republic of China); National Taiwan University, Taipei, Taipei, Taiwan (Republic of China); Chung Shan Medical University, Taichung, Taichung, Taiwan (Republic of China); National Taiwan Normal University, Taipei, Taipei, Taiwan (Republic of China)

## Abstract

**Background:**

Never-married women constitute a significant yet understudied segment of Taiwan’s older adult population. Unlike their married counterparts, these women deliberately chose independence and careers over traditional marriage paths. Using the Gendered Life Course Perspective, this study examines how these early-life choices shape their aging trajectories and welfare needs.

**Methods:**

This qualitative study employed semi-structured in-depth interviews with 12 purposively sampled participants from diverse sources, ensuring variation in age and educational background. Data were analyzed using thematic analysis to identify key patterns and themes.

**Findings:**

Never-married older women’s experiences reveal distinct patterns in redefining aging without traditional family support. 1. Deliberate autonomy reshaping independence: Participants intentionally remained unmarried, prioritizing careers and personal independence over societal expectations, fundamentally restructuring their aging experience. 2. Self-sufficiency as adaptive strategy: Without spousal or child support, these women developed enhanced resilience that enables them to navigate aging with greater self-determination. 3. Specialized welfare and community needs: Unlike married older adults, these women prioritize government-supported housing, formal advance care planning mechanisms, and senior communities. They express strong preferences for living arrangements that provide social opportunities and life support systems while maintaining independent living with the convenience and security of community environments—services that strategically substitute for family-based support.

**Conclusions and Implications:**

Never-married older women’s deliberate life choices shape unique aging experiences and welfare needs. Policies should respond with targeted housing programs, community-based supports, and advance care planning mechanisms that substitute for family assistance—acknowledging how these women redefine aging through independence while addressing structural service gaps.

